# Traffic Volatility Forecasting Using an Omnibus Family GARCH Modeling Framework

**DOI:** 10.3390/e24101392

**Published:** 2022-09-29

**Authors:** Jishun Ou, Xiangmei Huang, Yang Zhou, Zhigang Zhou, Qinghui Nie

**Affiliations:** 1 College of Architectural Science and Engineering, Yangzhou University, Yangzhou 225127, China; 2 State Key Laboratory of Mechanics and Control of Mechanical Structures, Nanjing University of Aeronautics and Astronautics, Nanjing 210016, China; 3 Zachry Department of Civil and Environmental Engineering, Texas A&M University, College Station, TX 77840, USA

**Keywords:** traffic volatility, asymmetric property, omnibus family GARCH model, short-term traffic flow forecasting, traffic reliability

## Abstract

Traffic volatility modeling has been highly valued in recent years because of its advantages in describing the uncertainty of traffic flow during the short-term forecasting process. A few generalized autoregressive conditional heteroscedastic (GARCH) models have been developed to capture and hence forecast the volatility of traffic flow. Although these models have been confirmed to be capable of producing more reliable forecasts than traditional point forecasting models, the more or less imposed restrictions on parameter estimations may make the asymmetric property of traffic volatility be not or insufficiently considered. Furthermore, the performance of the models has not been fully evaluated and compared in the traffic forecasting context, rendering the choice of the models dilemmatic for traffic volatility modeling. In this study, an omnibus traffic volatility forecasting framework is proposed, where various traffic volatility models with symmetric and asymmetric properties can be developed in a unifying way by fixing or flexibly estimating three key parameters, namely the Box-Cox transformation coefficient λ, the shift factor b, and the rotation factor c. Extensive traffic speed datasets collected from urban roads of Kunshan city, China, and from freeway segments of the San Diego Region, USA, were used to evaluate the proposed framework and develop traffic volatility forecasting models in a number of case studies. The models include the standard GARCH, the threshold GARCH (TGARCH), the nonlinear ARCH (NGARCH), the nonlinear-asymmetric GARCH (NAGARCH), the Glosten–Jagannathan–Runkle GARCH (GJR-GARCH), and the family GARCH (FGARCH). The mean forecasting performance of the models was measured with mean absolute error (MAE) and mean absolute percentage error (MAPE), while the volatility forecasting performance of the models was measured with volatility mean absolute error (VMAE), directional accuracy (DA), kickoff percentage (KP), and average confidence length (ACL). Experimental results demonstrate the effectiveness and flexibility of the proposed framework and provide insights into how to develop and select proper traffic volatility forecasting models in different situations.

## 1. Introduction

Short-term travel flow forecasting serves as an essential tool to provide useful traffic flow information in the immediate future, and hence wide supports to intelligent transportation systems (ITSs), especially advanced traveler information system, route guidance system, and proactive traffic signal control system. For instance, with accurate real-time traffic flow forecasts, a proactive signal control system is able to set up strategies to significantly reduce traffic delay, and a route guidance system is capable of providing travelers with the optimal route with a minimum travel cost. With this in mind, plenty of methods have been proposed for short-term traffic flow forecasting, which can be generally divided into two categories: (1) traditional forecasting methods that concentrate on improving the accuracy of traffic mean forecasts; and (2) traffic volatility forecasting that focus on the unpredictable part to capture the time-dependent variances.

During the last couple of decades, extensive traffic flow forecasting studies have been conducted using time series models [1,2,3,4], Kalman filtering [5,6], nonparametric regression [7,8], neural networks [9,10], Bayesian network [11,12], random forests [13,14], deep neural networks [15,16,17], and so on. The majority of the methods belong to point forecasting methods, in which the second moment (i.e., variance) of the traffic variables was presumed as a constant. Therefore, traffic volatility was artificially omitted during the forecasting process.

As the traffic reliability or uncertainty problem has been highly valued in recent years, many volatility models have been proposed for short-term traffic flow forecasting. Compared with the point forecasting models that presume a constant variance, the volatility models can not only produce accurate mean forecasts but also give more reliable forecasting confidence intervals by capturing the temporal evolution of conditional variances. Among the volatility models, the GARCH-type models, such as the fractionally integrated asymmetric power ARCH (FIAPARCH) model [18], the exponential GARCH (EGARCH) model [19], and the GJR-GARCH model [20], have enjoyed much popularity in past years, for their outstanding ability to describe the asymmetric effect of positive and negative shocks on volatility. Even though these models have been confirmed to be able to produce more reliable forecasts than the traditional point forecasting models, the more or less imposed restrictions on parameter estimations may make the asymmetric property of traffic volatility be not or insufficiently considered. Furthermore, the performance of the models has not been fully evaluated and compared in the traffic forecasting context, rendering the choice of the models dilemmatic for traffic volatility modeling. Therefore, it is necessary to develop a flexible traffic volatility forecasting framework under which various volatility models can be easily implemented and fully tested to yield more reliable forecasts and enhance the forecasting models’ applicability.

In this research, an omnibus traffic volatility forecasting framework based on the FGARCH model [21] is proposed to model traffic speed volatility on urban roads and freeways. The volatility models under the proposed framework are established by fixing or flexibly estimating three key parameters, namely the Box-Cox transformation coefficient λ, the shift factor b, and the rotation factor c. Extensive case studies show that the proposed framework can be used to account for both shift and rotation asymmetric effects and allows for more flexible traffic volatility descriptions that are usually restricted in some classical volatility models. The achieved findings provide insights into how to develop and select proper traffic volatility forecasting models in different situations and are helpful for building traffic flow forecasting models with high reliability and good applicability.

The main contributions of this research include two aspects. First, we present a comprehensive traffic volatility forecasting framework, which can be utilized to develop effective volatility models in a flexible manner, and are capable of properly capturing the evolution of conditional variance with symmetric or asymmetric properties. Second, we conduct extensive experimental studies to fully evaluate and compare the performance of the established volatility forecasting models, including the symmetric standard GARCH [22] and the asymmetric TGARCH [23], NGARCH [24], NAGARCH [25], GJR-GARCH [19], and FGARCH [21] and further summarize their applicability in various situations.

The remainder of this paper is organized as follows: In Section 2, a comprehensive literature review regarding traffic volatility forecasting is provided. Then, the proposed framework based on the FGARCH model for traffic volatility forecasting is elaborated in Section 3. In Section 4, the performance of the developed traffic volatility forecasting models is fully evaluated and compared using real traffic speed datasets collected from urban road links and freeway segments. Conclusions and future work are summed up in Section 5.

## 2. Literature Review

During the past decades, a large number of volatility methods have been developed for short-term traffic flow forecasting. In general, these methods describe the traffic volatility during the forecasting process by using the standard GARCH or its advanced extensions.

The GARCH-type models were firstly proposed in the fields of finance and economics. In order to deal with the problem that the volatility of financial time series changes over time, the autoregressive conditional heteroscedastic (ARCH) model was introduced by Engle [26], and then further promoted by Bollerslev [22] as the GARCH model. In a following study, Kamarianakis et al. [27] pointed out that the time-varying nature of traffic volatility can be recognized as the alternative large movement period that can be observed when the traffic condition changes. Therefore, a standard GARCH model was applied to study the volatility of relative speed. Given the strong ability to capture the traffic volatility with time-variant conditional variances, the GARCH model has been widely applied in many pieces of research for short-term traffic flow forecasting. Guo and Williams [28] proposed to use the autoregressive moving average (ARMA) model for traffic speed mean forecasting and the GARCH model for traffic speed volatility forecasting. At the stage of online processing, a layered adaptive Kalman filter approach was developed. Similarly, Chen et al. [29] proposed an integrated model which combines the autoregressive integrated moving average (ARIMA) model and the GARCH model for traffic flow forecasting and reached the conclusion that the time-variant confidence intervals achieved can provide more reliable information for travelers, compared with the standard ARIMA model. Ding et al. [30] modeled the dynamic volatility of the subway short-term ridership forecasting process by constructing four kinds of integrated ARIMA-GARCH models. Their experimental results show the performance of the hybrid models is better than the traditional models because of the improvement in the reliability of the forecasting point value with the associated coverage probability of the forecasting interval. Considering that the inherent relationships between different traffic variables could contribute to the improvement of traffic forecasting accuracy and reliability, Xia et al. [31] developed a multivariate GARCH model for traffic volume and speed volatility forecasting, in which a vector autoregressive model was used for jointly forecasting traffic volume and speed mean values. In addition, from the perspective of traffic flow decomposition, Chen et al. [32] proposed a time-series analysis and supervised-learning (TSA-SL) model for short-term traffic flow prediction by decomposing the traffic flow series into two components, namely periodicity and volatility.

Due to the standard GARCH process having a linear relationship between the conditional variance and the shocks in the past, the GARCH model is limited in perfectly describing the temporal evolution of traffic volatility. Moreover, the squared term in the standard GARCH model does not allow the asymmetric effects of traffic volatility that might exist when traffic condition changes dramatically. Therefore, Tsekeris and Stathopoulos [18] presented a fractionally integrated asymmetric power ARCH (FIAPARCH) model to relax the linear and symmetric restrictions of the standard GARCH model, and in return, to better represent the nonlinear and asymmetric properties of traffic volatility. In their research, the fractionally integrated component was introduced to describe the long memory of traffic volatility since the standard GARCH process can only capture the short memory of the volatility evolution that was restrained by the assumption of exponentially decayed propagation of shocks. Evaluation results show that the FIAPARCH model outperforms the standard GARCH model in traffic volatility forecasting. Similarly, Zhang et al. [19] applied another two asymmetric volatility models, i.e., an EGARCH model and a GJR-GARCH model, for reliable short-term travel time forecasting. Compared with the standard GARCH model, both the EGARCH and GJR-GARCH models allow the conditional variance to respond differently to the past negative and positive shocks that release the symmetric restriction in the GARCH model. Performance evaluation demonstrates that the GJR-GARCH is better than the standard GARCH model and the EGARCH model in terms of the forecasting bandwidth and coverage probability.

Considering traffic volatility may be better modeled by removing underlying traffic patterns that can be decomposed from original traffic flow data series, Zhang et al. [20] proposed a hybrid traffic flow forecasting model that used spectral analysis to remove the periodic trend of traffic flows using an ARIMA process to model the deterministic component of traffic flow, and using a GJR-GARCH process to model the traffic volatility. Experimental results show that the hybrid model is able to improve forecasting accuracy and reliability. Years later, Lin et al. [33] proposed a hybrid PSO-ELM model to quantify the uncertainty of border crossing traffic volume prediction and compared it with the adaptive Kalman filter method used by Guo et al. [28] and the GJR-GARCH method proposed by Zhang et al. [20]. The results confirm that the proposed PSO-ELM model can always reach satisfactory forecasting reliability. As a nonlinear analytical model, the integer-valued GARCH (INGARCH) can be utilized to capture the characteristics of network traffic. Therefore, it was introduced in the modeling of network traffic forecasting by Kim [34] and turned out to be more competitive than the ARIMA, GARCH, and long short-term memory neural network. In addition, Yao et al. [35] presented a nonlinear hybrid method for traffic flow forecasting, which uses the ARIMA-GARCH model to predict the similar and volatile parts, and applies the Markov model with state membership degree and wavelet neural network to predict the irregular part. Another hybrid model named ARIMA-GARCH-M developed by Lin and Huang [36] is able to predict short-term high-speed traffic flow data with good accuracy and reliability. To extend the GARCH model to be used for spatial data, Sato and Matsuda [37] proposed a spatial GARCH (S-GARCH) model. Considering the effect of both space and time restrictions, Hølleland and Karlsen [38] introduced a stationary spatiotemporal GARCH. To deal with the time-varying characteristic of traffic flow, a hybrid method that combines the ARIMA, maximum correntropy criterion, conditional kernel density estimation, and GARCH model was put forward by Zhao et al. [39], which outperforms the ARIMA model in terms of both accuracy and reliability.

The aforementioned GARCH-type models describe the evolution of traffic volatility from linear to nonlinear, from symmetric effect to asymmetric effect. Though the dynamics of traffic volatility has been modeled explicitly step-by-step, the ability of capturing traffic volatility may be subject to the restrictions of the appointed GARCH-type models. For instance, the asymmetric property of volatility can be captured by EGARCH, GJR-GARCH, and asymmetric power ARCH models because of the introduced “rotation factor” in the ARCH term. However, another critical factor—“shift factor”—that can also be used to describe the asymmetric property is not considered in these models. According to Helbing et al. [40,41], the volatile behavior dynamics of drivers was found to be similar to the stockholders in a financial market. As both the rotation and shift effects have been recognized in the financial market [21], it is reasonable and necessary to develop a more flexible volatility model that permits both shift and rotation asymmetric properties for traffic volatility modeling. Moreover, according to Hentschel [21], multiple volatility models are able to be established within the same theoretical GARCH framework. However, the performance of these models on traffic volatility has not been comprehensively tested, making their feasibility in traffic reliability inexplicable for researchers and practitioners.

## 3. Method

**Figure 1:**
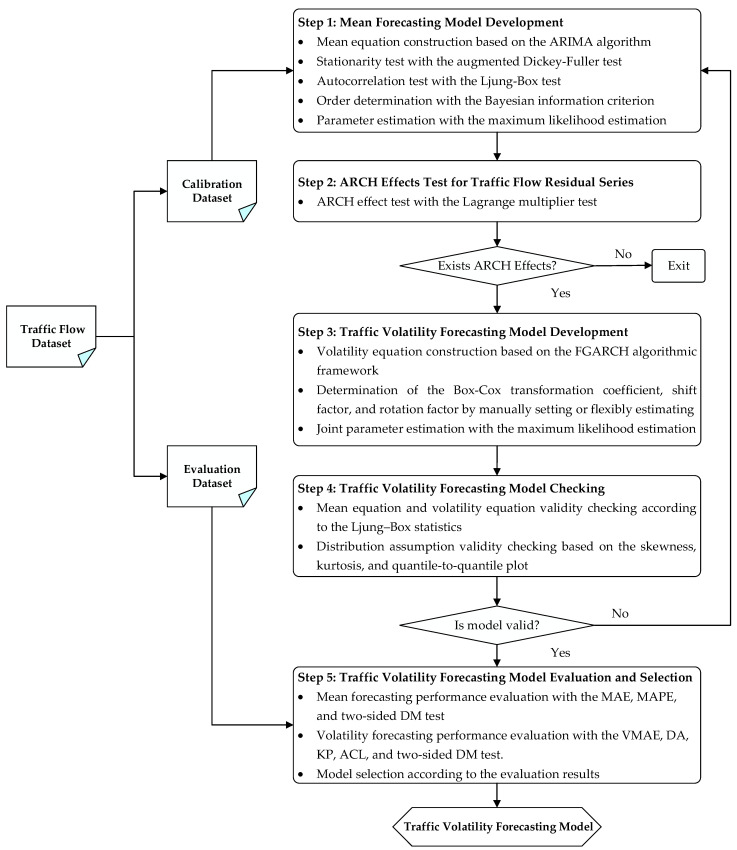
The proposed framework for traffic volatility forecasting.

To develop an effective traffic volatility model that can better capture the evolution of the conditional heteroskedasticity underlying traffic flow time series, an omnibus traffic volatility forecasting framework is presented, as depicted in Figure 1.

Generally, the framework can be systematically deconstructed into five steps. First, a mean forecasting model is built based on a calibration dataset and the ARIMA algorithm. To achieve satisfactory mean forecasts, the stationarity test and autocorrelation test are conducted using the augmented Dickey–Fuller (ADF) test [42] and the Ljung–Box test [43], respectively, and the orders of the ARIMA model are optimally identified with a step-wise algorithm [44] based on the Bayesian information criterion (BIC) [45]. Second, conditional heteroscedasticity, also known as the ARCH effect, is checked for the residual series obtained according to the mean equation. To achieve this purpose, the Lagrange multiplier test [26] is carried out. Third, if the ARCH effects are statistically significant, multiple traffic volatility models based on the FGARCH algorithmic framework are established to capture the symmetric or asymmetric effects in traffic volatility, while a joint estimation of the mean and volatility equations is performed to determine the model parameters using the maximum likelihood estimation (MLE) [46] method. Fourth, the developed models are checked to ensure the validity of the mean equation, volatility equation, as well as the distribution assumption. Finally, the traffic volatility forecasting models are evaluated in terms of performance measures of both mean forecasting and volatility forecasting, and the best model is selected for different application scenarios. In what follows, each step of the proposed framework is described in more details.

### 3.1. Mean Forecasting Model Development

The purpose of developing the mean forecasting model is to remove the sample mean from the traffic flow data and obtain the residual series for further traffic volatility modeling. One of the most widely used mean forecasting models, namely ARIMA [19,20,27,31], can be applied to fit the mean values of the traffic flow time series. Note that if the modeling traffic flow data contains the seasonal components, the seasonal ARIMA (SARIMA) [3,4] model can be constructed to ensure good mean forecasting performance. In this study, we only use one day’s data to calibrate the model, where the seasonality is not a typical characteristic of the traffic flow time series. As a result, the ARIMA model is established, which is mathematically expressed as

(1) $$\phi_{p}\left(B\right){\left(1-B\right)}^{d}x_{t}=\theta_{q}\left(B\right)\varepsilon_{t},$$

where $x_{t}$ is the traffic variable at time t, B is the backshift operator, d is the differencing order, p is the autoregressive polynomial order, q is the moving average polynomial order, ϕ and θ are the autoregressive and moving average parameters, and $\varepsilon_{t}$ is the error term. After the specification of the mean equation, four further subprocedures need to be carried out to establish the qualified mean forecasting model.

First, the stationarity of the traffic flow time series needs to be checked, which is an important assumption in modeling the ARIMA model. The ADF test [42] is a unit root test, which can be utilized to check the stationarity of a time series. The presence of a unit root implies the analyzed time series is non-stationary and the number of unit roots corresponds to the number of differencing operations required to make the series stationary. For the traffic flow time series $\left\{x_{t}\right\}$, to verify the existence of a unit root in an AR (p), we can perform the test $H_{0}:\beta =1$ versus $H_{1}:\beta <1$ with an ADF regression

(2) $$x_{t}=c_{t}+\beta x_{t-1}+\emptyset_{1}∆x_{t-1}+\cdots +\emptyset_{p-1}∆x_{t-\left(p-1\right)}+\varepsilon_{t},$$

where $c_{t}$ is a deterministic function of the time index t, and can be a constant or a linear function of t, $\emptyset_{1},\ldots \emptyset_{p-1}$ are the autoregressive coefficients, $∆x_{j}=x_{j}-x_{j-1}$ is the differenced series of $x_{t}$, β is the coefficient of $x_{t}$. Equation (2) can also be rewritten as

(3) $$∆x_{t}=c_{t}+\beta_{c}x_{t-1}+\emptyset_{1}∆x_{t-1}+\cdots +\emptyset_{p-1}∆x_{t-\left(p-1\right)}+\varepsilon_{t},$$

where $\beta_{c}=\beta -1$ and the equivalent hypothesis can be replaced as $H_{0}:\beta_{c}=0$ versus $H_{1}:\beta_{c}<0$. If we accept $H_{0}$ means the traffic flow series needs to be differenced to make it stationary, otherwise the stationary condition is satisfied.

Second, the autocorrelation of the traffic flow time series is tested with the Ljung–Box test [43]. As a linear time series model, ARIMA is characterized by its autocorrelation function (ACF), and its modeling process makes use of the sample ACF to specify the forecasting model that can capture the dynamic dependence of the data. In view of this, it is necessary to check whether several autocorrelations of the series $\left\{x_{t}\right\}$ are 0. The Ljung-Box test is a popular statistical means to achieve the above goal. The test statistic is given as

(4) $$Q\left(m\right)=n\left(n+2\right)\sum_{i=1}^{m}\frac{{\hat{\rho }}_{i}^{2}}{n-i},$$

where m is the autoregressive lag order of the series $\left\{x_{t}\right\}$ and can be commonly set as $\ln\left(n\right)$, n is the sample size, ${\hat{\rho }}_{i}$ is the sample ACF of $\left\{x_{t}\right\}$ and a biased estimate of $\rho_{i}$ that is the lag-i autocorrelation of $\left\{x_{t}\right\}$. The null hypothesis is $H_{0}:\rho_{1}=\cdots =\rho_{m}=0$ and the alternative hypothesis is $H_{1}:\rho_{i}\neq 0$ for $i\in \left\{1,\;\ldots ,\;m\right\}$. If $Q\left(m\right)>\chi_{m}^{2}\left(\alpha \right)$, $H_{0}$ will be rejected, where $\chi_{m}^{2}\left(\alpha \right)$ denotes the $100\left(1-\alpha \right)\mathrm{th}$ percentile of a chi-squared distribution with m degrees of freedom.

Third, a step-wise algorithm proposed by Hyndman and Khandakar [44] is introduced to determine the autoregressive order p and moving average order q. One of the advantages of the algorithm is that it is able to automatically identify the appropriate orders of the mean equation and guarantee a valid model. This advantage is significant since the order p and order q are often application-dependent, implying the modeling process may be labor-intensive or time-consuming if we determine the orders manually with site-to-site traffic flow data. In this study, the BIC [45] is adopted to measure the quality of each candidate model during the step-wise searching procedure and is defined as

(5) $$BIC\left(\tau \right)=ln\left({\tilde{\sigma }}_{\tau }^{2}\right)+\frac{\tau ln\left(n\right)}{n},$$

where ${\tilde{\sigma }}_{\tau }^{2}$ is the maximum likelihood estimate of $\sigma_{x}^{2}$ that is the variance of $\left\{\varepsilon_{t}\right\}$, $ln\left(n\right)$ is the penalty of each parameter. The selection rule is to compute $BIC\left(\tau \right)$ for τ=0, . . . , ρ, where ρ is a prespecified positive integer, and select the value of τ as the order p or q that has the minimum BIC value.

Once the orders of the ARIMA are optimally determined, the last subprocedure of building the mean forecasting model is to estimate the parameters ϕ and θ with the MLE method. Note that this subprocedure is optional since we can estimate the parameters of the mean equation and volatility equation jointly. In this study, we conduct this subprocedure because we will test whether the joint estimation procedure can indeed improve the point forecasting accuracy. Interested readers can refer to [47] for more details.

### 3.2. ARCH Effects Test for Traffic Flow Residual Series

Once the mean forecasting model is established, the residual series can be computed according to the observations and mean forecasts. The conditional heteroscedasticity of the residual series, also known ARCH effects, needs to be carefully checked to model the traffic volatility. In view of this, the Lagrange multiplier test [26] is introduced to check whether there are ARCH effects in the traffic flow residual series. The Lagrange multiplier test is to conduct testing in the following regression:

(6) $$\varepsilon_{t}^{2}=\alpha_{0}+\alpha_{1}\varepsilon_{t-1}^{2}+\cdots +\alpha_{m}\varepsilon_{t-m}^{2}+e_{t},t=m+1,\ldots ,n,$$

where $\varepsilon_{t}$ is the residual of the mean equation at time t, $e_{t}$ denotes the regression error term. More specifically, we define the null hypothesis as $H_{0}:\;\alpha_{1}=\cdots =\alpha_{m}=0$ against the alternative hypothesis as $H_{1}:\alpha_{i}\neq 0$ for some i between 1 and m. Denote $SSR_{0}=\sum_{t=m+1}^{n}{\left(\varepsilon_{t}{}^{2}-\bar{\omega }\right)}^{2}$, where $\omega =\frac{1}{n}\sum_{t=1}^{n}\varepsilon_{t}{}^{2}$ is the sample mean of $\varepsilon_{t}{}^{2}$, and $SSR_{1}=\sum_{t=m+1}^{n}{\hat{e}}_{t}{}^{2}$, where $\;{\hat{e}}_{t}$ is the least squares residual of the prior linear regression. Then we have

(7) $$F=\frac{\left(SSR_{0}-SSR_{1}\right)/m}{SSR_{1}/\left(n-2m-1\right)},$$

which follows an F distribution with degrees of freedom m and n−2m−1 under $\;H_{0}$. When n is sufficiently large, mF can be used as the test statistic, which is asymptotically a chi-squared distribution with m degrees of freedom under the null hypothesis. Let $\chi_{m}^{2}\left(\alpha \right)$ be the upper $100\left(1-\alpha \right)\mathrm{th}$ percentile of $\;\chi_{m}^{2}$. If $mF>\chi_{m}^{2}\left(\alpha \right)$, we reject the null hypothesis and the residual series is heteroscedastic; otherwise, it does not have any ARCH effect. In this study, if the P-values computed from the F statistic are less than the significant level α=0.05, a proper volatility model needs to be established to describe the evolution of the traffic flow volatility.

### 3.3. Traffic Volatility Forecasting Model Development

When the significant ARCH effects are checked, the subsequent step is to capture the effects with a proper volatility model. The ARCH model proposed by Engle [26] provides the first systematic framework for volatility modeling. Although owning some advantages for volatility modeling, the ARCH model also has some weaknesses. For example, the ARCH model is rather restrictive and often imposes complicated constraints with the increase of the orders, resulting in the limited applicability of the model. The GARCH model, first introduced by Bollerslev [22], is a useful extension of the ARCH model and can provide a simpler parametric function to describe the volatility evolution. It should be indicated that both models respond equally to the positive and negative shocks [47], and hence cannot be utilized to describe the asymmetric property of the volatility, a common phenomenon in a vast number of application areas.

To deal with the above issue, various GARCH-type models have been presented. Among the GARCH-type models for short-term traffic flow forecasting, the GARCH (1,1) model has been verified as the most adequate and simple representation of traffic volatility [11,18,31]. Therefore, the omnibus FGARCH (1,1) based volatility equation is constructed in this study for short-term traffic volatility forecasting, which is mathematically expressed as

(8) $$\left\{\begin{array}{l} \varepsilon_{t}=z_{t}\sigma_{t}\\ z_{t}\sim IIN\left(0,1\right)\\ \sigma_{t}^{\lambda }=\xi +\delta \sigma_{t-1}^{\lambda }f{\left(\varepsilon_{t}\right)}^{\lambda }+\eta \sigma_{t-1}^{\lambda }\\ f\left(\varepsilon_{t}\right)=\left|\varepsilon_{t}-b\right|-c\left(\varepsilon_{t}-b\right) \end{array}\right.,$$

where $\varepsilon_{t}$ is the error term of the mean equation, $\sigma_{t}$ is the time-variant conditional standard deviation to reflect the traffic volatility, random variable $z_{t}$ is a strong white noise process, ξ, δ, and η are the parameters that need to be estimated, λ is the Box-Cox transformation coefficient, $f\left(\varepsilon_{t}\right)=\left|\varepsilon_{t}-b\right|-c\left(\varepsilon_{t}-b\right)$ is the shifted and rotated absolute value function, in which the parameter b controls the shift effect and the parameter c governs the rotation effect.

The parameter λ can be obtained from Box-Cox transformation for the conditional standard deviation. Therefore, the relationship between the conditional variances and all shocks in the past may be more flexible than the linear relationship. As indicated by Ding et al. [48], a better estimate of the power transformation coefficient λ can help maintain the long memory property of the volatility. In the standard GARCH model, λ is fixed as 2, which only describes the short memory property of volatility. The shift factor b and the rotation factor c are introduced to model the asymmetric impact upon volatility exerted by positive and negative shocks—called the leverage effect in the financial markets. Small shocks are dominated by the shift effect, while the rotation is more important for large shocks [21]. As Helbing et al. [40] demonstrated, there are intrinsic similarities between market players and traffic network users, implying that the shift and rotation effects of the asymmetric property that have been recognized in financial volatility may also exist in traffic volatility.

The above volatility equation has a flexible form for modeling volatility by controlling the parameters λ, b, and c. For instance, we can derive the standard GARCH model by restricting the parameters λ=2 and b=c=0. That is, the standard GARCH model imposes restrictive nonnegative constraints on the parameters and also restricts a linear relationship between conditional variances and all shocks in the past. Moreover, the restriction b=c=0 makes the standard GARCH model a symmetric model, in which both the shift and rotation effects are not allowed to describe the asymmetric property of volatility evolution. Similar restrictions can be seen in other GARCH-type models such as λ=0, b=0 in the EGARCH model, and b=0 and $\left|c\right|\leq 1$ in the APARCH and TGARCH models. By manually setting up or freely estimating part or all of the three parameters, we can further obtain multiple models that can capture different traffic flow volatility characteristics. Interested readers can refer to Hentschel [21] for the detailed restrictions of the three parameters in the FGARCH algorithmic framework. Overall, by constructing the FGARCH-based volatility equation, we can implement multiple traffic volatility forecasting models within a unifying framework, and are herein, able to provide a more appropriate and flexible form for explicitly describing the evolution of volatility and to fully investigate the feasibility of the models in different situations. The parameters of the developed traffic volatility forecasting models are finally determined by using the MLE method [46].

### 3.4. Traffic Volatility Forecasting Model Checking

The adequacy of the fitted models needs to be examined carefully before traffic volatility forecasting. More specifically, we need to check the validity of the established mean equation and volatility equation. The validity of the mean equation can be examined by applying the Ljung–Box test to the standardized residual series $\left\{\varepsilon_{t}/\sigma_{t}\right\}$ and the validity of the volatility equation can be checked by applying the Ljung–Box test to the squared standardized residual series $\left\{{\left(\varepsilon_{t}/\sigma_{t}\right)}^{2}\right\}$. When the above P-values of the Ljung–Box statistic are smaller than $\chi_{m}^{2}\left(\alpha \right)$, the traffic volatility forecasting model can reduce the heteroscedasticity in the residual series and capture the statistical characteristics of traffic volatility well. Moreover, the skewness, kurtosis, and quantile-to-quantile plot of the standardized residual series can be applied to examine the validity of the distribution assumption [47].

### 3.5. Traffic Volatility Forecasting Model Evaluation and Selection

The last step of the framework is to evaluate the performance of the developed models and select the most effective one for further traffic volatility forecasting according to the evaluation results.

For traffic flow mean forecasting performance evaluation, two typical measures, i.e., the mean absolute error (MAE) and the mean absolute percentage error (MAPE), are selected, which are defined as follows.

(9) $$\mathrm{MAE}=\frac{1}{n}\overset{n}{\underset{t=1}{\sum }}\left|x_{t}-{\hat{x}}_{t}\right|$$

(10) $$\mathrm{MAPE}=\frac{1}{n}\overset{n}{\underset{t=1}{\sum }}\left|\frac{x_{t}-{\hat{x}}_{t}}{x_{t}}\right|$$

In the above equations, $x_{t}$ is the observed value of the traffic variable at time t, ${\hat{x}}_{t}$ is the forecast value of the traffic variable at time t, and n is the number of the tested data samples. To check the difference of the MAE measures of the two competing models, the two-sided DM test statistic [49] is adopted.

With regard to traffic volatility forecasting performance evaluation, four classical measures, including the kickoff percentage (KP), the average confidence length (ACL), the volatility MAE (VMAE), and the directional accuracy, are employed. For short-term traffic flow forecasting, the KP measure indicates the probability that future observations do not lie in the forecasted confidence intervals when given the evaluation sample size. Theoretically, the better the performance is, the closer the KP is at a given significant level α (α=0.05 in this study) [19]. The ACL measure quantifies the average length of the forecasted confidence interval. To reduce forecasting uncertainties, the forecasted confidence interval should be reasonably as narrow as possible. According to Chen et al. [50], VMAE measures the average magnitude of volatility forecasting error, and DA measures the correctness of the turning point forecasts that gives a rough indication of the average direction of the forecast volatility. The four measures are defined as follows.

(11) $$\mathrm{KP}=\frac{1}{n}\overset{n}{\underset{t=1}{\sum }}K_{t}\times 100\%$$

(12) $$\mathrm{ACL}=\frac{1}{n}\sum_{t=1}^{n}CL_{t}\;=\frac{1}{n}\sum_{t=1}^{n}\left(\hat{U}\left(x_{t}\right)-\hat{L}\left(x_{t}\right)\right)$$

(13) $$\mathrm{VMAE}=\frac{1}{n}\overset{n}{\underset{t=1}{\sum }}\left|u_{t}^{2}-{\hat{u}}_{t}^{2}\right|$$

(14) $$\mathrm{DA}=\frac{1}{n-1}\overset{n}{\underset{t=2}{\sum }}a_{t}$$

(15) $$a_{t}=\left\{\begin{array}{cc} 1 & \left(u_{t}^{2}-u_{t-1}^{2}\right)\left({\hat{u}}_{t}^{2}-{\hat{u}}_{t-1}^{2}\right)\geq 0\\ 0 & \mathrm{otherwise} \end{array}\right.$$

In the above equations, $K_{t}$ is a two-valued variable, $K_{t}=0$ if $x_{t}\in \left[\hat{L}\left(x_{t}\right),\hat{U}\left(x_{t}\right)\right]$; otherwise, $K_{t}=1$. $\hat{L}\left(x_{t}\right)$ and $\hat{U}\left(x_{t}\right)$ are the lower and upper bounds of the forecasted confidence interval at time t. $CL_{t}$ is the forecasted confidence length at time t. $u_{t}$ and ${\hat{u}}_{t}$ represent the observed volatility and forecasted volatility, respectively. As the observed volatility is unknown in reality, $u_{t}^{2}={\left(x_{t}-{\bar{x}}_{t}\right)}^{2}$ can be used as the surrogate [50], where $\bar{x}$ is the mean value of the tested data samples. The forecasted volatility is quantified as ${\hat{u}}_{t}^{2}={\left(x_{t}-{\hat{x}}_{t}\right)}^{2}$.

## 4. Experimental Analysis

### 4.1. Data Description

The proposed traffic volatility forecasting framework and developed models were tested using real traffic speed data collected from urban roads of Kunshan city, China and freeway segments of the San Diego Region, USA [51]. To investigate the performance of the models under different traffic conditions, two kinds of datasets were constructed. One consists of the datasets collected under normal traffic conditions, while the other includes the datasets collected under incident conditions. The former datasets were collected at 35 stations on 5 types of roads, namely major arterials, minor arterials, branches, expressway, and freeway. For each station, we collected data in four days. Two of them are the weekdays, and the other two are weekends. For the latter, the traffic flow data was acquired from the freeway monitoring stations associated with 12 incidents occurred in different days. The traffic speed data of each station was aggregated at 5-min intervals and divided into a calibration dataset and an evaluation dataset. The calibration dataset was used to build the traffic volatility model, while the evaluation dataset was utilized to test the performance of the calibrated model. More detailed information on the used data is described in Appendix A Table A1 and Table A2. It is noted that the missing, erroneous, and suspicious data is less than 3% for each monitoring station during the data collection periods, ensuring that the selected traffic datasets are suitable for model calibration and evaluation.

### 4.2. Performance Evaluation of Mean Forecasting Models

To evaluate the mean forecasting performance of the developed models, the MAE and its two-sided DM test statistics were computed and compared. In this study, the parameters of the mean forecasting model can be estimated in two ways—namely separate estimation and joint estimation. The former is implemented based on a separate ARIMA modeling process, while the latter is achieved by estimating the parameters of the mean equation and volatility equation jointly. The two ways may lead to different mean forecasting performance. Therefore, it is necessary to investigate the performance difference between the two ways.

Table 1 shows the performance comparisons between the separate estimation and joint estimation on the evaluation datasets. The first column of the table indicates the traffic flow patterns considered in this research, including normal traffic patterns on weekdays, normal traffic patterns on weekends, and incident traffic patterns. The second column depicts the comparison results. The label “Win” means the joint estimation way outperforms the separate estimation way. The label “Lose” means the joint estimation way is worse than the separate estimation way. The label “Tie” means the two ways have identical or similar performance. The digital number in the table cell represents the number of stations associated with the label in the corresponding row. The percentage in the parentheses indicates the proportion of the associated label.

As can be seen from the table, under normal traffic conditions, the two estimation ways show very similar performance, especially on weekends. However, under incident traffic conditions, there were significant differences between the performance of the two estimation ways on nearly half of the evaluation stations. More specifically, the separate estimation way achieved better performance than the joint estimation way. This implies that it is better to first estimate the parameters of the mean equation and further use the residual time series to estimate the parameters of the volatility equation when modeling traffic volatility under incident traffic conditions.

Traffic on different types of roads exhibits distinct fluctuation patterns. To properly evaluate the performance of the established models under different volatility levels, we grouped the stations with different road types according to the calculated VMAE measure, as illustrated in Table 2. As seen, for normal conditions on weekdays, the traffic on minor arterial and branch show significant fluctuations and had a high volatility level. The major arterial and freeway possess medium volatility level, while the expressway has the lowest volatility. Different from the normal conditions on weekdays, the daily traffic on weekends passed through the freeway appears to have very low fluctuations and was hence put into the low volatility level group. The traffic flow on freeway segments under incident conditions shows medium-level volatility.

Table 3 provides the average MAE value and its DM test rank of the developed models on the evaluation datasets. As mentioned above, the stations were categorized into three groups (H-high volatility level; M-medium volatility level; L-low volatility level) according to the volatility level. The average MAE value was calculated as the mean value of all stations in the same group. The DM test rank was computed based on the following procedure: First, for each station in the same volatility level group, we compare the performance of two models according to the DM statistic and p value. If the p value is less than the significant level (0.05 in this study), it means one of the two comparative models is significantly better or worse than the other model. By repeating the above comparison process, we can obtain the rank of each model. Next, the ranks of each model are averaged for all stations in the same volatility level group. The smaller the rank is, the better performance the model possesses.

The best rank is labeled with the red color, while the worst rank is labeled with the blue color. Note that when the DM test ranks of two models are same, the best model is further determined by the average MAE values. As indicated by the average MAE values, the developed six models show very similar mean forecasting performance. By further going through the DM test ranks, we can find that the NGARCH has better performance when the volatility levels are high and medium. Comparatively, the TGARCH shows worse performance in the same situations. It is a bit surprising that the TGARCH model shows advantages over other models under incident conditions. We can also see that the freely estimated FGARCH model takes the most advantage at the high volatility level on weekdays.

Table 4 gives the average MAPE value and its rank for each developed model. The computing procedure is similar with that of the MAE measure. From the evaluation results, we can obtain a similar observation, further confirming the conclusions achieved above.

### 4.3. Performance Evaluation of Volatility Forecasting Models

To effectively evaluate the volatility forecasting performance of the developed models, the VMAE, DA, KP, and ACL measures were calculated. The four measures quantify the model performance from different perspectives. The VMAE measures the accuracy of traffic volatility forecasting. The DA gives the volatility direction accuracy of the forecasting models. KP and ACL ensure the forecasts fall into the predicted confidence interval as much as possible when the interval length is short enough. Considering the inconsistency of the measures in some cases, we also computed the ranks of the models for each measure to make a fair comparison.

**Figure 2:**
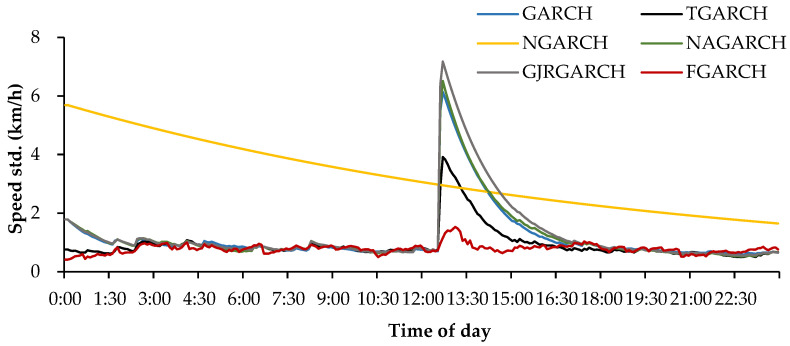
Comparison of forecasted traffic speed standard deviations of six developed models.

Table 5 lists the average VMAE value and its corresponding DM test rank of the developed models. As can be seen, the volatility forecasting performance is quite similar for the six models. However, their ranks indicate that different models usually show distinct advantages compared to different traffic speed datasets. As indicated by Hentschel [21], the GARCH-type models developed in this study show similar forecasting results on the Daily U.S. stock dataset due to the fact that they are derived from the same modeling framework. Our experimental results also point to a similar conclusion, although the models indeed show distinct forecasting behaviors on several datasets. For instance, Table 6 and Figure 2 depict the distinct performance of the models on the incident dataset I818_1118078.

As seen in Table 3, the estimated parameters of the mean equation of the models appear to be very similar because the orders of the equation are the same. For the volatility equation, it is obvious that the estimated values of the parameter λ exhibit distinct diversity among different models. The different values of the parameter λ implies that the developed models may capture the conditional variance and respond differently to the past shocks when modeling the same traffic flow series. In addition, both of the freely estimated parameters b and c are different from 0, indicating the existence of asymmetric property of traffic speed volatility controlled by the shift and rotation effects at the same time. Moreover, for the FGARCH model, the estimated parameter b is positive and parameter c is negative, implying that the asymmetric property of traffic speed volatility at this station may be dominated by the combination of rightward shift effect and anticlockwise rotation effect, which is consistent with the “news impact curve” introduced by Pagan and Schwert [52].

**Figure A1:**
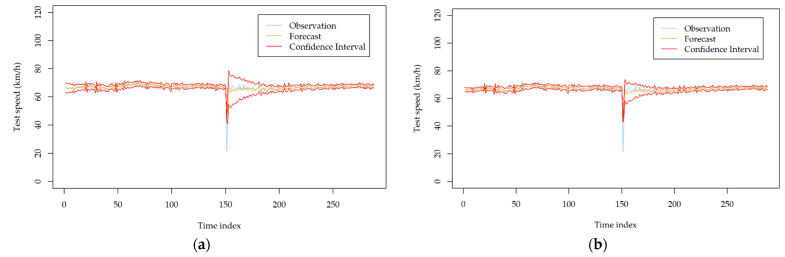

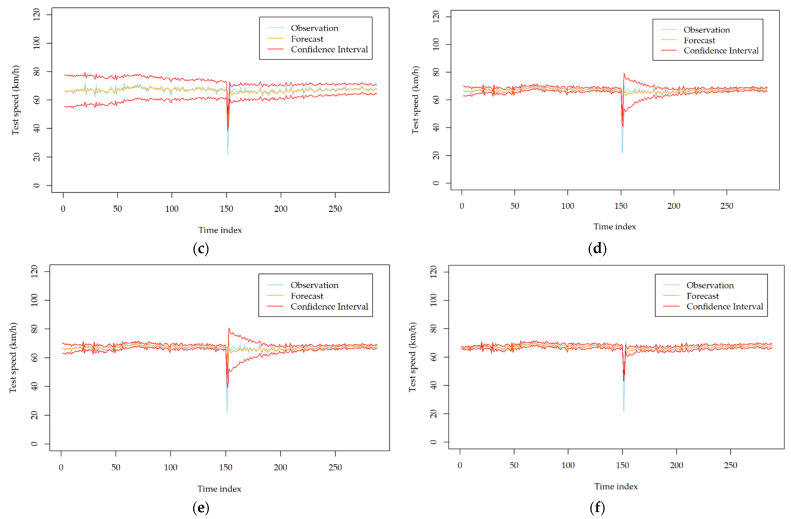
Volatility forecasting results of the developed models with the traffic speed dataset collected at station I818_1118078 on 10 August 2010; (**a**) GARCH; (**b**) TGARCH; (**c**) NGARCH; (**d**) NAGARCH; (**e**) GJR-GARCH; (**f**) FGARCH.

Figure 2 depicts the forecasted speed standard deviation curves of the developed models. As seen from the figure, these models respond differently to the fluctuations induced by the incident. The NGARCH model decreases the standard deviation forecasts gradually with the time evolution, while the other five models are all able to capture the incident volatility but respond with different magnitudes. To further check the effectiveness of the models, the observation, mean forecasts, as well as the forecast confidence intervals are given in Appendix A Figure A1. It can easily be seen that the FGARCH exhibits the best volatility forecasting performance among the six models because it can not only correctly capture the volatility characteristics caused by the incident but also provide more reliable and narrower confidence interval than the others.

The DA measure describes the forecasting directional accuracy of the given model. In view of this, we calculated the average DA value and its rank of the six models, as shown in Table 7. From the table, it is obvious that under normal traffic conditions, the standard GARCH model has the best performance on weekdays and the NAGARCH model shows more advantages over other models on weekends. Interestingly, the FGARCH has the best performance under incident conditions while not performing well under normal traffic conditions.

The absolute deviation of KP from the significance level of 0.05 was calculated to check the validity of the models at each traffic station. The calculation results include the average value and its rank on all stations of the same volatility level group, which are illustrated in Table 8. From the table, we can see that the standard GARCH model and the GJR-GARCH model show better performance than the other four models in terms of KPD, which means the two models exhibit the smallest absolute deviation of KP from the given significant level (α=0.05) and tried to make the confidence interval contain forecasted data samples as much as possible. In contrast, the FGARCH exhibits unsatisfactory performance in most cases.

The average ACL value and the rank of each developed model are presented in Table 9. As can be seen from the table, the overall ACL values of the models show similarities in different situations. Relatively, the standard GARCH model, GJR-GARCH model, and FGARCH model tend to enlarge the forecasted confidence interval. In contrast, the TGARCH model attempts to give a narrower confidence interval in most cases. It needs to be indicated that it is commonly difficult for a volatility model to achieve the lowest KP and ACL at the same time because the two measures are counterbalanced in describing volatility characteristics.

### 4.4. Impact of Identifying Orders of ARIMA on Forecasting Performance

There are commonly two strategies for identifying the orders of the mean equation of the proposed framework. The first is an automtic identification strategy, which uses the step-wise searching algorithm [44] with an information criteria (e.g., AIC or BIC). The other is a manual identification strategy that finds the optimal orders by manually inspecting the ACF and PACF plots. Each strategy has its own advantages. For example, the automatic identification strategy is more computationally efficient and can be utilized to handle extensive datasets in an automated manner and hence realize significant cost savings, while the manual identification strategy is capable of providing more reliable forecasts in some cases.

**Figure 3:**
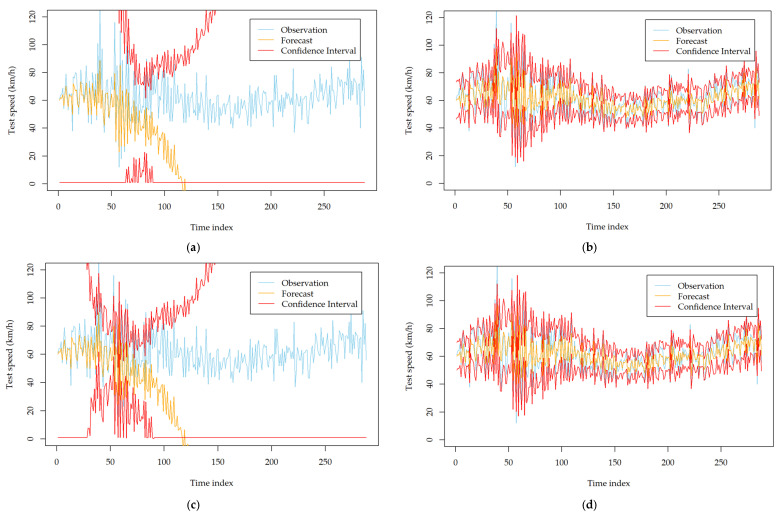

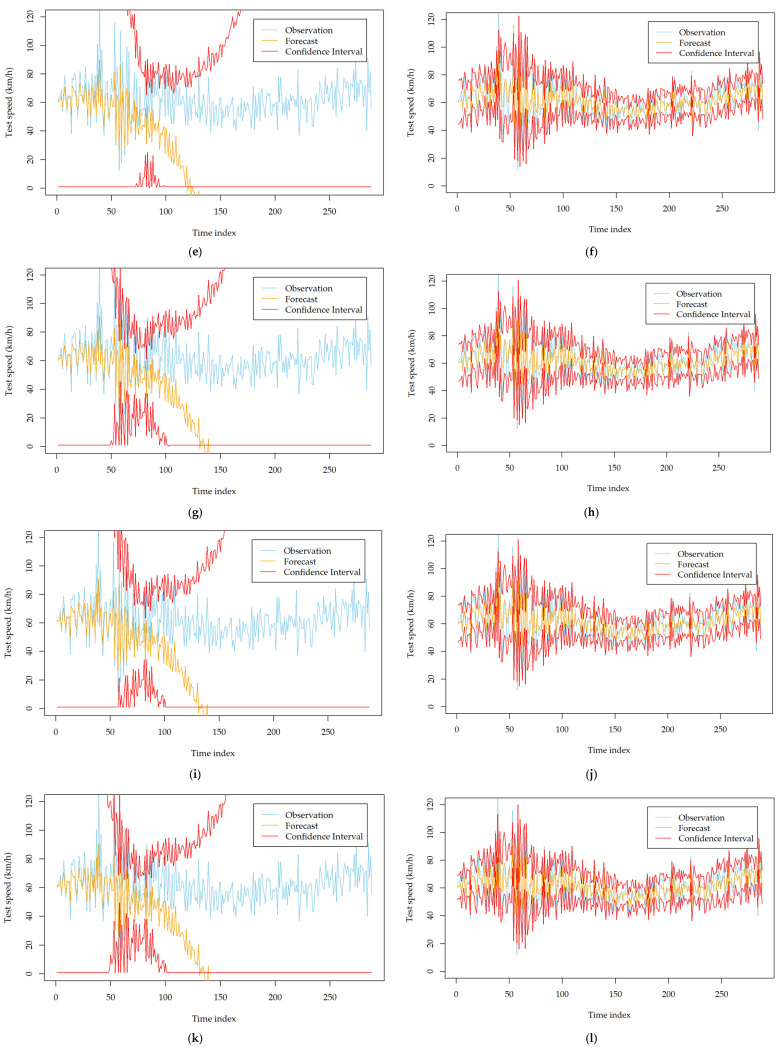
Comparison of the volatility forecasting performance of six developed models with different order identification strategies at station MA_1014014 on 9 September 2018; (**a**) GARCH (automatic order identification); (**b**) GARCH (manual order identification); (**c**) TGARCH (automatic order identification); (**d**) TGARCH (manual order identification); (**e**) NGARCH (automatic order identification); (**f**) NGARCH (manual order identification); (**g**) NAGARCH (automatic order identification); (**h**) NAGARCH (manual order identification); (**i**) GJR-GARCH (automatic order identification); (**j**) GJR-GARCH (manual order identification); (**k**) FGARCH (automatic order identification); (**l**) FGARCH (manual order identification).

To investigate the impact of the two order identification strategies on the forecasting performance of the developed models, we recorded the forecasting results using the two strategies. After careful inspection and calibration, we found that the two strategies lead to similar forecasting performance for most stations. However, for several specific monitoring stations, they show significant differences. Appendix A Table A3 and Table A4 show the comparison results on such kinds of stations. From the tables, we can see that the models established based on the automatic strategy can result in a significantly poor mean forecasting performance and volatility forecasting performance. The MAE and DA deteriorate dramatically for all of the developed models. This observation can be further obtained with Figure 3, which describes the forecasting results of the developed models at station MA_1014014 on 9 September 2018. The mean forecasts and volatility forecasts biase the true observations significantly, while they are improved evidently after using the manual order identification strategy. Therefore, it is better to carefully check the identified orders of ARIMA when all of the developed volatility models perform poorly and try to determine the orders with the manual strategy.

## 5. Conclusions

Traffic volatility forecasting plays a key role in traffic reliability or uncertainty analysis. Many GARCH-type models have been developed to capture volatility in the fields of finance and economics. However, the ability of these models to capture traffic volatility has not been fully investigated. In this study, a systematic traffic volatility forecasting framework based on an omnibus family GARCH model is proposed. Under this framework, six volatility models were developed by manually setting up or freely estimating three key parameters. The performance of the models was comprehensively evaluated and compared by using 47 traffic speed data collected from five kinds of roads under normal traffic conditions and incident conditions. A number of key findings were achieved based on the experimental results, which can provide insights into traffic volatility modeling for researchers and practitioners.

The experimental results in this research reveal the potential of explicitly modeling volatility evolution properties in improving traffic volatility forecasting performance. In the future, more traffic flow datasets could be used to evaluate the effectiveness of the proposed framework. The physical explanations about how the asymmetric property of traffic volatility is associated with various traffic phenomena are also worth exploring.

## Figures and Tables

**Table 1:** Mean forecasting performance comparisons of separate estimation and joint estimation.

Pattern	Result	Joint Estimation vs. Separate Estimation					
		S	T	N	NA	GJR	F
Normal (Weekday)	Win	3	4	4	3	3	5
		(8.57%)	(11.43%)	(11.43%)	(8.57%)	(8.57%)	(14.29%)
	Lose	3	7	5	3	7	4
		(8.57%)	(20.00%)	(14.29%)	(8.57%)	(20.00%)	(11.43%)
	Tie	29	24	26	29	25	26
		(82.86%)	(68.57%)	(74.29%)	(82.86%)	(71.43%)	(74.29%)
	Total	35	35	35	35	35	35
Normal (Weekend)	Win	3	3	2	2	2	4
		(8.57%)	(8.57%)	(5.71%)	(5.71%)	(5.71%)	(11.43%)
	Lose	3	1	1	3	1	1
		(8.57%)	(2.86%)	(2.86%)	(8.57%)	(2.86%)	(2.86%)
	Tie	29	31	32	30	32	30
		(82.86%)	(88.57%)	(91.43%)	(85.71%)	(91.43%)	(85.71%)
	Total	35	35	35	35	35	35
Incident	Win	2	3	3	2	2	2
		(16.67%)	(25.00%)	(25.00%)	(16.67%)	(16.67%)	(16.67%)
	Lose	5	3	3	3	5	5
		(41.67%)	(25.00%)	(25.00%)	(25.00%)	(41.67%)	(41.67%)
	Tie	5	6	6	7	5	5
		(41.67%)	(50.00%)	(50.00%)	(58.33%)	(41.67%)	(41.67%)
	Total	12	12	12	12	12	12

S (Standard GARCH), T (TGARCH), N (NGARCH), NA (NAGARCH), GJR (GJR-GARCH), F (FGARCH).

**Table 2:** Volatility level of the traffic speeds collected from different types of roads.

Road Type	Volatility under Normal Conditions (Weekday)	Volatility under Normal Conditions (Weekend)	Volatility under Incident Conditions
Major Arterial	126.529 (Medium)	96.567 (Medium)	
Minor Arterial	158.048 (High)	164.377 (High)	
Branch	135.374 (High)	129.801 (High)	
Expressway	6.014 (Low)	5.232 (Low)	
Freeway	129.723 (Medium)	5.691 (Low)	54.772 (Medium)

**Table 3:** Average MAE value and its DM test rank of the developed models on evaluation datasets.

MAE	GARCH Type	Normal (Weekday)			Normal (Weekend)			Incident
		H	M	L	H	M	L	
Value	S	4.241	2.867	0.980	4.249	3.422	0.757	1.222
	T	4.251	3.461	0.998	4.244	3.426	0.771	1.149
	N	4.246	2.705	0.982	4.244	3.422	0.760	1.165
	NA	4.241	2.868	1.010	4.250	3.424	0.760	1.208
	GJR	4.238	2.877	1.003	4.247	3.426	0.856	1.222
	F	4.322	2.728	0.987	4.257	3.427	0.856	1.169
Rank	S	1.643	1.400	1.000	1.714	1.000	2.417	2.917
	T	2.143	1.733	1.500	1.786	1.556	2.417	2.417
	N	1.643	1.067	1.833	1.286	1.000	2.250	2.833
	NA	1.786	1.467	2.500	1.857	1.000	1.750	2.667
	GJR	1.714	1.733	1.667	1.286	1.000	2.500	3.250
	F	1.429	1.267	2.167	1.714	1.000	1.833	3.083

S (Standard GARCH), T (TGARCH), N (NGARCH), NA (NAGARCH), GJR (GJR-GARCH), F (FGARCH).

**Table 4:** Average MAPE value and its rank of the developed models on evaluation datasets.

MAPE	GARCH Type	Normal (Weekday)			Normal (Weekend)			Incident
		H	M	L	H	M	L	
Value	S	14.865	9.231	1.392	14.456	8.316	1.095	2.464
	T	14.924	10.133	1.425	14.478	8.324	1.114	2.383
	N	14.881	8.988	1.397	14.429	8.313	1.099	2.383
	NA	14.884	9.220	1.440	14.504	8.316	1.097	2.431
	GJR	14.819	9.271	1.430	14.441	8.318	1.234	2.458
	F	15.121	9.039	1.405	14.528	8.314	1.235	2.384
Rank	S	2.929	2.667	1.333	3.214	2.111	2.750	2.500
	T	3.143	3.267	1.667	2.786	2.889	2.167	2.417
	N	2.714	2.933	2.000	2.500	1.444	2.500	2.333
	NA	2.429	1.933	3.000	3.357	1.778	1.500	2.417
	GJR	2.357	2.600	2.000	2.714	2.333	2.167	3.083
	F	2.286	3.067	2.667	3.000	2.222	2.083	3.000

S (Standard GARCH), T (TGARCH), N (NGARCH), NA (NAGARCH), GJR (GJR-GARCH), F (FGARCH).

**Table 5:** Average VMAE value and its DM test rank of the developed models on evaluation datasets.

VMAE	GARCH Type	Normal (Weekday)			Normal (Weekend)			Incident
		H	M	L	H	M	L	
Value	S	152.915	127.988	6.061	154.412	96.573	5.462	58.940
	T	152.703	264.279	6.006	154.398	96.561	5.459	56.690
	N	152.912	111.636	6.061	154.493	96.574	5.457	56.560
	NA	152.909	128.103	6.013	154.168	96.551	5.450	59.080
	GJR	153.084	127.614	6.015	154.407	96.512	5.652	58.970
	F	153.489	111.735	6.051	154.226	96.623	5.754	56.560
Rank	S	2.071	1.667	1.000	1.786	1.556	1.667	1.250
	T	1.500	2.067	1.000	1.929	1.333	1.667	2.170
	N	2.214	1.867	1.000	2.000	1.556	1.583	1.330
	NA	2.071	2.200	1.000	1.429	1.444	1.917	1.670
	GJR	2.214	1.867	1.000	1.929	1.222	1.917	1.670
	F	2.286	2.133	1.000	1.357	1.556	2.750	1.830

S (Standard GARCH), T (TGARCH), N (NGARCH), NA (NAGARCH), GJR (GJR-GARCH), F (FGARCH).

**Table 6:** Joint parameter estimation of the mean equation and volatility equation for six developed models with the traffic speed dataset collected at station I818_1118078 on 10 August 2010.

Mean Equation		Volatility Equation		
ARIMA (2,1,1)	$\emptyset_{1}$ = 0.454 $\emptyset_{2}$ = 0.001 $\theta_{1}$ = −0.903	GARCH (1,1)	ξ = 0.024 δ = 0.057 η = 0.904	λ = 2.00 b = 0.00 c = 0.00
	$\emptyset_{1}$ = 0.458 $\emptyset_{2}$ = −0.006 $\theta_{1}$ = −0.890	TGARCH (1,1)	ξ = 0.022 δ = 0.073 η = 0.916	λ = 1.00 b = 0.00 c = 0.32
	$\emptyset_{1}$ = 0.457 $\emptyset_{2}$ = −0.008 $\theta_{1}$ = −0.905	NGARCH (1,1)	ξ = 0.003 δ = 0.000 η = 0.983	λ = 4.00 b = 0.00 c = 0.00
	$\emptyset_{1}$ = 0.451 $\emptyset_{2}$ = −0.008 $\theta_{1}$ = −0.896	NAGARCH (1,1)	ξ = 0.016 δ = 0.057 η = 0.905	λ = 2.00 b = 0.49 c = 0.00
	$\emptyset_{1}$ = 0.452 $\emptyset_{2}$ = 0.001 $\theta_{1}$ = −0.902	GJR-GARCH (1,1)	ξ = 0.018 δ = 0.055 η = 0.914	λ = 2.00 b = 0.00 c = 0.19
	$\emptyset_{1}$ = 0.435 $\emptyset_{2}$ = 0.006 $\theta_{1}$ = −0.891	FGARCH (1,1)	ξ = 0.104 δ = 0.072 η = 0.825	λ = 0.04 b = 0.87 c = −0.58

**Table 7:** Average DA value and its rank of the developed models on evaluation datasets.

DA	GARCH Type	Normal (Weekday)			Normal (Weekend)			Incident
		H	M	L	H	M	L	
Value	S	0.681	0.632	0.853	0.687	0.685	0.711	0.600
	T	0.682	0.625	0.845	0.691	0.687	0.709	0.604
	N	0.679	0.634	0.853	0.685	0.689	0.712	0.602
	NA	0.680	0.634	0.839	0.694	0.688	0.718	0.602
	GJR	0.679	0.636	0.845	0.684	0.688	0.716	0.599
	F	0.669	0.638	0.844	0.688	0.686	0.710	0.604
Rank	S	2.000	2.133	1.333	2.786	2.778	3.167	2.667
	T	2.071	3.333	2.667	2.714	2.556	3.333	2.750
	N	3.500	2.533	1.333	3.000	1.889	3.583	2.500
	NA	2.643	2.867	4.833	2.000	1.778	2.333	2.333
	GJR	2.143	2.667	2.667	3.643	1.889	2.250	2.583
	F	3.643	3.067	2.833	2.714	3.667	3.250	2.333

S (Standard GARCH), T (TGARCH), N (NGARCH), NA (NAGARCH), GJR (GJR-GARCH), F (FGARCH).

**Table 8:** Average KPD value and its rank of six developed models on evaluation datasets.

KPD	GARCH Type	Normal (Weekday)			Normal (Weekend)			Incident
		H	M	L	H	M	L	
Value	S	0.010	0.007	0.012	0.011	0.011	0.009	0.008
	T	0.013	0.008	0.018	0.012	0.013	0.011	0.034
	N	0.012	0.007	0.168	0.011	0.014	0.011	0.110
	NA	0.014	0.009	0.016	0.015	0.011	0.011	0.028
	GJR	0.010	0.006	0.014	0.012	0.009	0.012	0.010
	F	0.089	0.077	0.014	0.022	0.019	0.017	0.031
Rank	S	2.286	2.600	2.500	2.429	2.333	2.583	1.583
	T	3.286	3.867	4.000	2.571	3.000	2.750	3.417
	N	2.500	2.800	3.000	2.429	3.000	3.250	4.750
	NA	3.857	2.600	3.333	3.214	2.778	3.417	2.250
	GJR	2.500	2.200	2.833	2.286	1.889	2.917	2.083
	F	3.500	4.200	3.500	4.929	5.000	3.917	3.583

S (Standard GARCH), T (TGARCH), N (NGARCH), NA (NAGARCH), GJR (GJR-GARCH), F (FGARCH).

**Table 9:** Average ACL value and its rank of six developed models on evaluation datasets.

ACL	GARCH Type	Normal (Weekday)			Normal (Weekend)			Incident
		H	M	L	H	M	L	
Value	S	21.789	13.995	5.193	21.354	16.974	3.897	6.833
	T	22.423	15.343	5.113	21.137	16.864	3.952	4.867
	N	21.992	13.447	4.245	21.303	17.044	3.846	5.772
	NA	21.749	13.954	5.122	21.576	16.949	3.870	6.324
	GJR	22.024	14.017	5.072	21.388	16.931	4.163	6.783
	F	21.554	13.389	5.313	22.021	16.794	4.113	7.856
Rank	S	3.429	4.067	3.833	3.214	3.222	3.833	3.833
	T	3.571	4.333	2.667	2.500	3.222	2.917	2.250
	N	3.214	2.600	3.500	3.714	4.000	3.250	3.250
	NA	2.786	3.333	3.500	3.500	3.333	2.833	3.583
	GJR	3.857	3.533	2.833	3.786	3.889	3.500	3.750
	F	4.071	2.733	4.167	4.143	2.444	3.667	3.417

S (Standard GARCH), T (TGARCH), N (NGARCH), NA (NAGARCH), GJR (GJR-GARCH), F (FGARCH).

## Data Availability

Not applicable.

## Appendix A

**Table A1:** Traffic flow datasets collected under normal traffic conditions.

Station ID	Road Type	Calibration Dataset		Evaluation Dataset	
		Period	Sample Size	Period	Sample Size
MA_1004015	Major Arterial	4 September 2018 8 September 2018	288 288	5 September 2018 9 September 2018	288 288
MA_1004027					
MA_1004028					
MA_1012021					
MA_1014005					
MA_1014009					
MA_1014014					
MA_1014018					
MA_1016012					
MI_1003001	Minor Arterial	4 September 2018 8 September 2018	288 288	5 September 2018 9 September 2018	288 288
MI_1004019					
MI_1005012					
MI_1005013					
MI_1005015					
MI_1005016					
MI_1005018					
MI_1006019					
MI_1006020					
B_1004017	Branch	4 September 2018 8 September 2018	288 288	5 September 2018 9 September 2018	288 288
B_1013001					
B_1013003					
B_1015003					
B_1015005					
E_10131301	Expressway	4 September 2018 8 September 2018	288 288	5 September 2018 9 September 2018	288 288
E_10131501					
E_10132601					
E_10141101					
E_10141201					
E_10141401					
F_1108452	Freeway	2 March 2010 6 March 2010	288 288	3 March 2010 7 March 2010	288 288
F_1108498					
F_1114254					
F_1114735					
F_1114806					
F_1117755					

**Table A2:** Traffic flow datasets collected under incident conditions.

Station ID	Road Type	Calibration Dataset		Evaluation Dataset	
		Period	Sample Size	Period	Sample Size
I190_1117774	Freeway	25 July 2010	288	26 July 2010	288
I224_1117755	Freeway	25 July 2010	288	26 July 2010	288
I275_1117835	Freeway	8 April 2010	288	9 April 2010	288
I371_1114806	Freeway	24 September 2010	288	25 September 2010	288
I373_1108711	Freeway	8 October 2010	288	9 October 2010	288
I383_1115355	Freeway	4 April 2010	288	5 April 2010	288
I393_1108615	Freeway	19 June 2010	288	20 June 2010	288
I689_1118537	Freeway	15 July 2010	288	16 July 2010	288
I772_1117835	Freeway	31 July 2010	288	1 August 2010	288
I818_1118078	Freeway	10 August 2010	288	11 August 2010	288
I914_1117809	Freeway	2 September 2010	288	3 September 2010	288
I1039_1118078	Freeway	6 October 2010	288	7 October 2010	288

**Table A3:** Comparison of the MAE measure of the developed models with different order identification strategies.

Station	Test Period	Strategy	$\left(p,d,q\right)$	MAE (veh/5 min)					
				S	T	N	NA	GJR	F
MA_1014014	9 September 2018	Automatic	(2,1,3)	1297.99	1298.40	962.04	554.66	582.07	522.14
		Manual	(0,1,2)	5.02	5.03	5.02	5.02	5.02	5.04
MI_1005012	9 September 2018	Automatic	(3,1,3)	173.48	3.20	13.22	386.48	9.60	33.73
		Manual	(0,1,2)	3.19	3.20	3.18	3.18	3.19	3.18
E_10141201	5 September 2018	Automatic	(2,0,2)	4.62	8.16	1.91	4.31	4.25	1.96
		Manual	(0,1,1)	1.05	1.18	1.05	1.21	1.20	1.06
I818_1118078	11 August 2010	Automatic	(2,0,1)	2.23	6.25	1.45	2.24	2.25	125.03
		Manual	(0,1,1)	0.76	0.76	0.75	0.76	0.76	0.76

S (Standard GARCH), T (TGARCH), N (NGARCH), NA (NAGARCH), GJR (GJR-GARCH), F (FGARCH).

**Table A4:** Comparison of the DA measure of the developed models with different order identification strategies.

Station	Test Period	Strategy	$\left(p,d,q\right)$	DA					
				S	T	N	NA	GJR	F
MA_1014014	9 September 2018	Automatic	(2,1,3)	0.5505	0.5679	0.5784	0.5505	0.5540	0.5505
		Manual	(0,1,2)	0.7666	0.7561	0.7700	0.7666	0.7666	0.7491
MI_1005012	9 September 2018	Automatic	(3,1,3)	0.4913	0.5923	0.5366	0.4948	0.5575	0.5331
		Manual	(0,1,2)	0.5854	0.5854	0.5958	0.5854	0.5889	0.5854
E_10141201	5 September 2018	Automatic	(2,0,2)	0.7944	0.7561	0.8118	0.8014	0.7944	0.7979
		Manual	(0,1,1)	0.8328	0.8014	0.8328	0.7979	0.8014	0.8328
I818_1118078	11 August 2010	Automatic	(2,0,1)	0.6028	0.6411	0.5923	0.6063	0.6028	0.5819
		Manual	(0,1,1)	0.7387	0.7387	0.7422	0.7352	0.7387	0.7282

S (Standard GARCH), T (TGARCH), N (NGARCH), NA (NAGARCH), GJR (GJR-GARCH), F (FGARCH).
